# Bacteriophage strain typing by rapid single molecule analysis

**DOI:** 10.1093/nar/gkv563

**Published:** 2015-10-10

**Authors:** Assaf Grunwald, Moran Dahan, Anna Giesbertz, Adam Nilsson, Lena K. Nyberg, Elmar Weinhold, Tobias Ambjörnsson, Fredrik Westerlund, Yuval Ebenstein

**Affiliations:** 1 Raymond and Beverly Sackler Faculty of Exact Sciences, School of Chemistry, Tel Aviv University, Tel Aviv 6997801, Israel; 2 Institute of Organic Chemistry, RWTH Aachen University, Aachen D-52056 Germany; 3 Department of Astronomy and Theoretical Physics, Lund University, Lund 223 62, Sweden; 4 Department of Biology and Biological Engineering, Chalmers University of Technology, Gothenburg 412 96, Sweden

## Abstract

Rapid characterization of unknown biological samples is under the focus of many current studies. Here we report a method for screening of biological samples by optical mapping of their DNA. We use a novel, one-step chemo-enzymatic reaction to covalently bind fluorophores to DNA at the four-base recognition sites of a DNA methyltransferase. Due to the diffraction limit of light, the dense distribution of labels results in a continuous fluorescent signal along the DNA. The amplitude modulations (AM) of the fluorescence intensity along the stretched DNA molecules exhibit a unique molecular fingerprint that can be used for identification. We show that this labelling scheme is highly informative, allowing accurate genotyping. We demonstrate the method by labelling the genomes of λ and T7 bacteriophages, resulting in a consistent, unique AM profile for each genome. These profiles are also successfully used for identification of the phages from a background phage library. Our method may provide a facile route for screening and typing of various organisms and has potential applications in metagenomics studies of various ecosystems.

## INTRODUCTION

Identification of microorganism populations in various substances, such as water, blood or soil, is of great importance for clinical and environmental studies (1,2). Currently, the leading approach for strain typing is metagenomics, where next generation sequencing methods (NGS) are used for partial or full sequencing of organism genomes, extracted directly from samples without prior culturing (3). NGS based metagenomics studies have in recent years contributed significantly to public health policies, ecosystem analysis and identification of new organisms (4,5). Nevertheless, this approach suffers from the same drawbacks as all NGS based methods, such as difficulties with facing unknown and previously unsequenced genomes, the need for relatively high amounts of initial genetic material, as well as high costs and tedious sample preparation and data analysis procedures. In addition, the short sequence reads, combined with the high similarity of different microbial genomes, hampers the ability of NGS to differentiate and quantify organisms in mixed populations (6). In the case of viruses and phages, which represent one of the most abundant entities in nature, metagenomic studies are facing even greater challenges. The high variability and rapid evolution of phages, combined with difficulties in viral culturing, complicates the process of sequencing and *de novo* assembly (7). For instance, even though it is known that ocean water contains large amounts of marine viruses (∼4·10^30^, most of them are phages) as of 2012 only 122 types of marine phages have been identified (8,9). Other typing methods, including partial polymerase chain reaction amplifications and sequencing of target genes, hybridization DNA arrays, pulsed-field gel electrophoresis for analysis of restriction fragments and phenotype based identification, suffer from similar drawbacks including problems with facing unknown mixed samples and difficulties in differentiating between similar species (10–12).

Single-molecule optical DNA mapping offers an alternative approach for genotyping long individual DNA molecules such as phage genomes. For optical mapping, fluorescent labels are attached to specific DNA sequences generating a unique fluorescent barcode that can be used for DNA identification, and further characterization of genomic information such as DNA damage or epigenetic modifications (13–21). One approach for generating sequence specific labels is to use enzymes for incorporation of fluorophores within the enzyme's recognition sites. This approach takes advantage of the efficiency and specificity of DNA processing enzymes, such as nicking enzymes (22,23) or DNA methyltransferases (MTase) (15–16,24–26), to catalyze the labelling reaction. After sequence specific labelling, DNA molecules are stretched out and the fluorescence barcode along them is recorded using single molecule imaging (27). DNA classification is done by identification of the pattern of fluorescent spots generated along the DNA and alignment of the molecules, to a reference or to each other, based on this pattern (13). This methodology has its roots in the first optical mapping experiments where gaps induced by restriction enzymes on stretched, surface immobilized DNA molecules were used for sequence alignment (28,29). More recently, the genome mapping approach was introduced by Kwok *et al.* (30,31). This approach utilizes nanochannel arrays to extend DNA molecules in solution and detect a pattern of isolated fluorescent spots generated by nick translation along the molecules.

**Figure 1.**
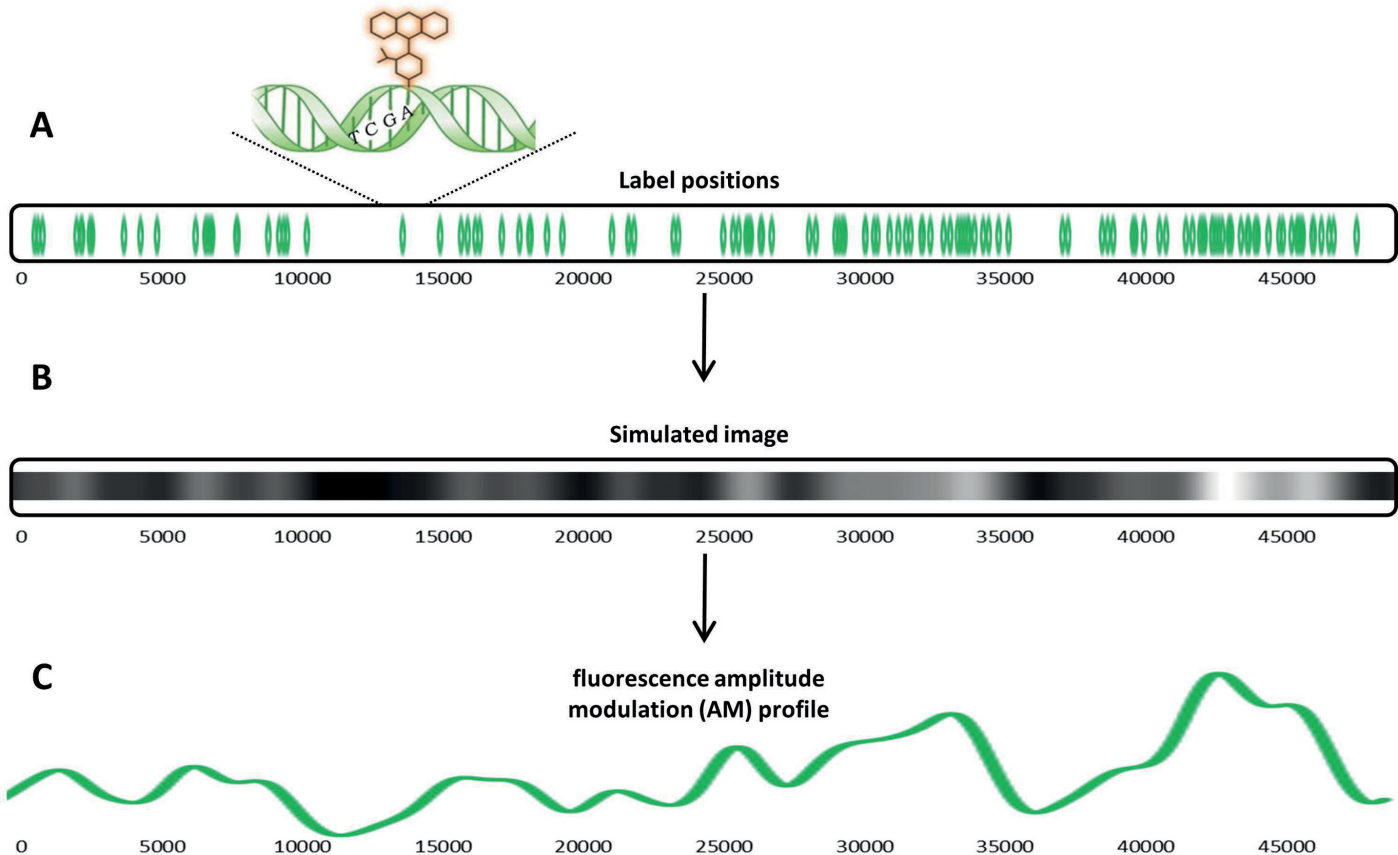
(**A**) The relative locations of M.TaqI labelling sites along λ-DNA. In total there are 121 sites on the 48.5 kbp long λ-DNA genome, each represented by a green mark. The length scale is given in bp below the cartoon. Above the molecule there is a ‘zoom-in’ representation of the labelling enzyme recognition site: TCGA and the fluorophore attached to the adenine base. (**B**) A simulated image showing the fluorescence intensity modulation along the image of a labelled λ-DNA genome (assuming that a point spread function spans 1500 bp). The total observed fluorescence is the convolution of overlapping point spread functions of neighbouring fluorophores. (**C**) A theoretical profile representing the expected amplitude modulations (AM) of a labelled λ-DNA genome under the same conditions as in (B).

**Scheme 1.**
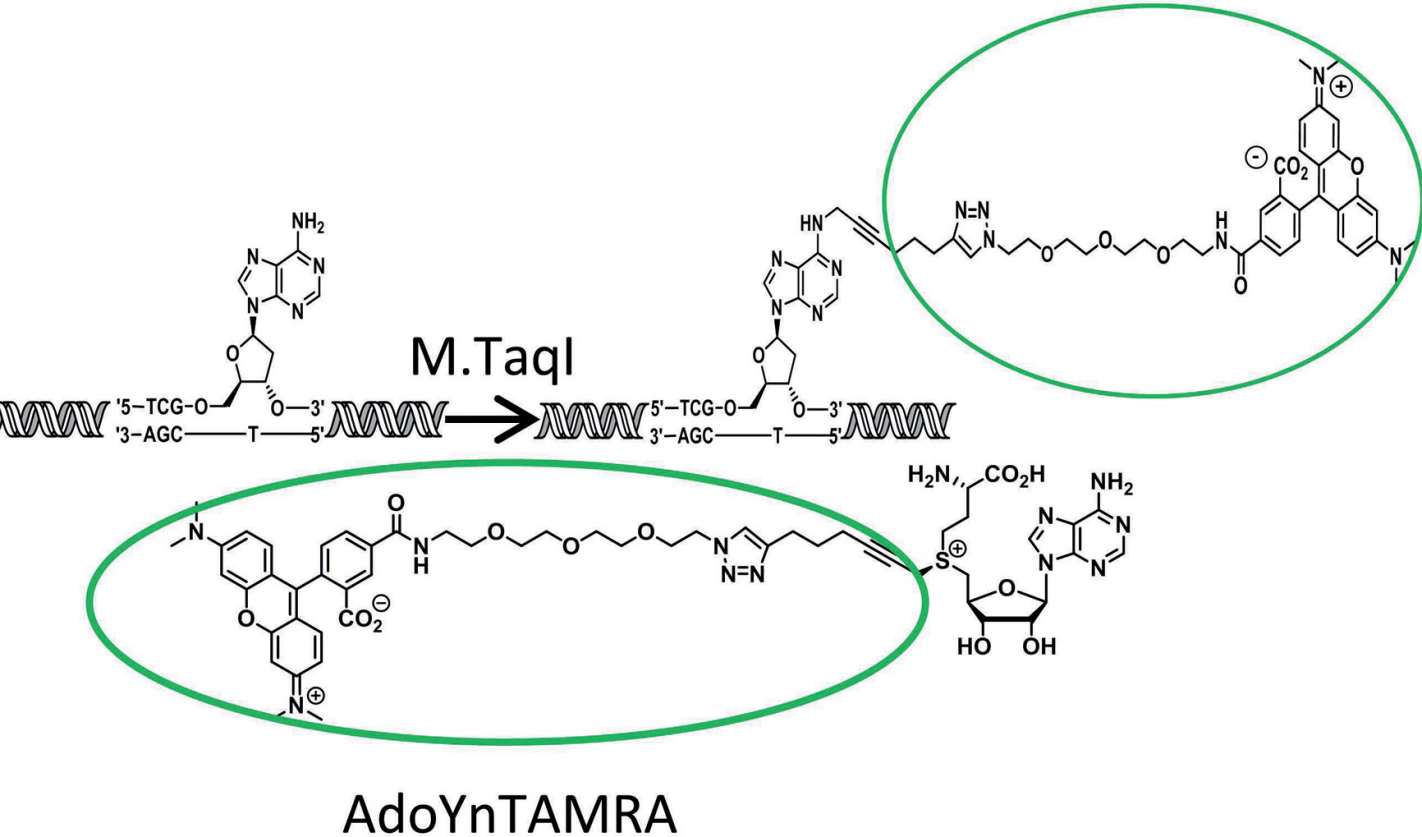
A representation of the labelling reaction: M.TaqI catalyzes the covalent transfer of the TAMRA fluorophore, together with an organic linker, from the synthetic co-factor-AdoYnTAMRA to an adenine residue that lies in M.TaqI's recognition sequence: TCGA.

Single-molecule imaging of long DNA molecules stretched in nanochannels is emerging as a valuable complement to DNA sequencing. It enables reading fluorescent tags along the DNA molecule and extracting genetic information from the specific fluorescence pattern. Bacteriophage genomes are relatively short and hence may be visualized intact, without the need for assembly, giving this approach an advantage over DNA sequencing for analysis of unknown or mixed samples. However, most reported mapping schemes fail with short genomes due to the difficulty of creating a unique pattern along short DNA molecules. A different approach for generating a fluorescent barcode along DNA is to deliberately generate a very dense pattern of labels that is not composed of isolated fluorescent spots. Instead, one can measure the amplitude modulations (AM) of fluorescence intensity along the DNA molecule (17–18,21). Each DNA sequence exhibits a unique label pattern and the intensity amplitude along the DNA is a convolution of proximal label intensities (overlapped due to the diffraction limit). This results in an AM profile that carries a unique ‘fingerprint’ for each underlying DNA molecule. The AM profile can be used for DNA identification without the need to resolve individual labels (Figure 1). Furthermore, since this approach does not require preliminary planning of the labelling pattern, it allows the use of a generic labelling procedure common to most DNA sequences without previous knowledge of the studied DNA. In this study we use a novel realization of the methyltransferase-directed labelling approach where a sequence specific DNA MTase is used to catalyze covalent attachment of a fluorophore to a DNA nucleotide within its recognition sequence (Martin *et al.*, under review) (Scheme 1). We use the DNA MTase M.TaqI, from *Thermus aquaticus* (32), to attach a fluorophore to the adenine residue in the four base pair sequence TCGA. Labelling is achieved in a single step by feeding the enzyme with a synthetic cofactor containing a fluorophore at the transfer position instead of the endogenous methyl group. The resulting label pattern is continuous due to the high frequency of the four-base labelling motif of this enzyme.

**Figure 2.**
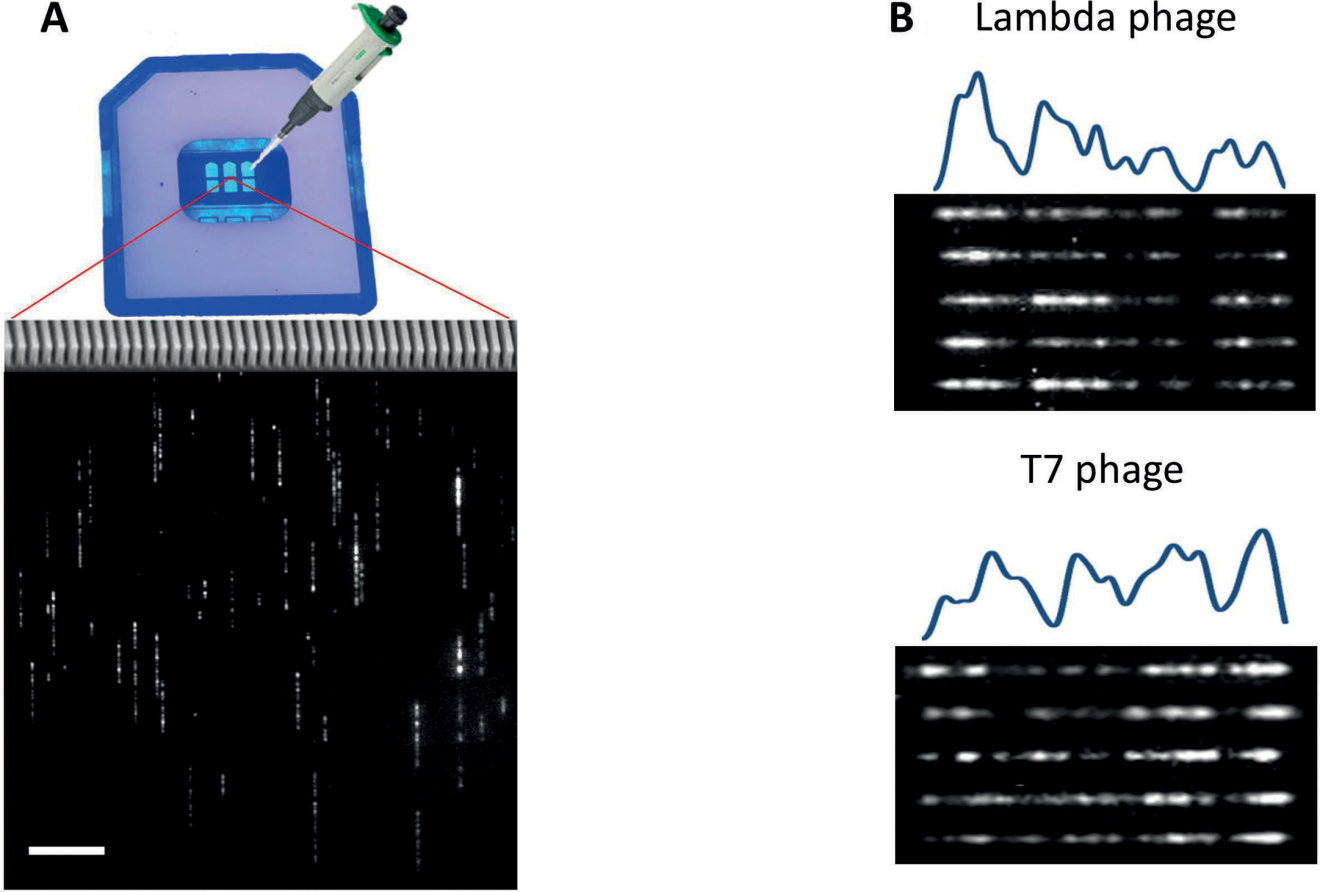
(**A**) On top, an image of a chip used in the experiments for stretching the labelled DNA. The chip contains three nanochannel arrays (red lines representing an enlargement of a part of the middle array). Each array has two reservoirs, an anterior and a posterior one (a cartoon of a pipette is pointing to the anterior reservoir to which the DNA is being loaded). On the bottom, an image of a field of view of the nanochannels containing stretched and labelled T7 genomes. The scale bar at the bottom left corner represents a distance of 10 μm. (**B**) Images of five representative λ (upper panel) and T7 (lower panel) genomes, labelled with M.TaqI and AdoYnTAMRA, and stretched in nanochannels. The molecules are aligned to each other and to the theoretical AM profile based on their similar AM profiles.

This labelling scheme is used together with DNA stretching in nanochannel arrays (Figure 2A) and cross-correlation analysis schemes to quickly identify short (∼50 kbp) bacteriophage genomes. We show that we are able to uniquely recognize these genomes from within a reference sequence containing multiple similar phages.

## MATERIALS AND METHODS

### DNA labelling and imaging

To generate sequence specific labelling we used the DNA MTase M.TaqI to catalyze the transfer of a carboxytetramethylrhodamine (TAMRA) fluorophore from the synthetic cofactor AdoYnTAMRA onto the adenine residue within its TCGA recognition sequence (32,33). A total of 1 μg of 48 kbp λ-DNA containing 121 M.TaqI sites or 39 kbp T7 DNA containing 111 M.TaqI sites (both from New England Biolabs, Ipswich MA, USA), was treated with 1.1 μg of M.TaqI and 40 μM of AdoYnTAMRA in labelling buffer (20 mM Tris/HOAc, 10 mM Mg(Cl)_2_, 50 mM KOAc, 1 mM DTT, pH 7.9) in a total reaction volume of 25 μl at 37°C for 1 h (Scheme 1). Protection-restriction assays were used to show that the efficiency of the labelling reaction is approaching 100% (Supplementary Figure S6). The labelled DNA was reacted with 100 μg of protein kinase K (Sigma-Aldrich, Rehovot, Israel) at 50°C for 1 h to disassemble protein and DNA aggregates. The reaction was cleaned by ethanol precipitation: 60 μl of cold absolute ethanol (Bio-Labs itd. Jerusalem, Israel) and 10 μl of sodium acetate 3M (Active-Motif, Carlsbad, CA, USA) were added to the reaction, the mixture was incubated for 30 min at −20°C followed by centrifugation at 19 k RCF for 30 min at 4°C. The pellet was washed in 70% absolute ethanol (Bio-Labs itd. Jerusalem, Israel) and dissolved in TE buffer to a final concentration of ∼50 ng/μl. Before imaging, the labelled DNA was stained with 0.5 μM of YOYO-1 (Invitrogen, Carlsbad, CA, USA) for visualization of its contour.

In order to stretch the DNA from its random coiled conformation into a linear form, we used silicon fabricated nanochannel arrays (∼45 nm square channels, BioNanoGenomics Inc., San Diego, CA, USA). An electric field was used to drive the DNA into the nanochannels; forcing it to stretch along them (Figure 2A). Labelled DNA (suspended in a flow buffer containing: 1.25 mM polyvinylpyrrolidone (Sigma-Aldrich, Rehovot, Israel), 3% of Tween-20 (Sigma-Aldrich, Rehovot, Israel) in 1/2× TBE) was loaded into reservoirs at the entrance of the nanochannels (34). An electric field was applied across the channels through electrodes immersed in the input and output reservoirs in a direction forcing the DNA into the channels. Once the DNA was stretched the voltage was stopped and the stretched DNA was imaged (Figure 2A).

The imaging setup consisted of an Olympus inverted microscope equipped with a 100X, NA=1.3 oil-immersion objective (Olympus), 473 nm, 50 mW, 532 nm, 200 mW, laser excitation sources (OEM lasers, USA) used, in combination with 510/20 and 580/60 filters, for imaging of the YOYO-1 and TAMRA labels accordingly and a Hamamatsu OrcaFlash 2.0 scientific CMOS camera for data acquisition (Hamamatsu Photonics Co., Hamamatsu, Japan). The silicon chip was fixed to an XY stage (Applied Scientific Instrumentation (ASI), Eugene, OR, USA) to allow scanning of the whole chip. The equipment and acquisition was controlled by μManager software (35).

### Data analysis

When studying AM profiles, the information is extracted from continuous modulations along the whole molecule rather than detecting single fluorescent spots. The overall intensity amplitude along a molecule is measured to generate an AM profile. A cross correlation (CC) is calculated between the experimental profile and a reference profile, generated from the known sequence. The output is a numerical value defining the degree of similarity between the two tested profiles. The extraction of AM from the data and the CC tests were done using a software developed to analyse intercalation based labelling (17–18,21,36). In our analysis we only considered molecules with a CC higher than 0.85 when compared to at least one of the theoretical sequences.

We also calculated the information score (IS) of our data. Simply put, the IS for a specific AM profiles is determined by the number of distinct features along the molecule and their modulation depth (i.e. the contrast). High IS indicates that the profile contains multiple, distinct peaks and valleys and thus its AM exhibits a unique fingerprint. As a result, the chance for false assignment of such profiles is relatively low. On the other hand, profiles of low IS, containing for instance only a single peak, can theoretically be assigned to various reference sequences with high confidence. Since this score is independent of the molecule length, high scores can both indicate a long molecule with spares features or a relatively short molecule with dense features. In the case of relatively short genomes, such as the phage genomes discussed here, it is essential to set an IS threshold for data filtration in order to obtain significant identification and reduce the computational burden of data analysis.

Nilsson *et al.* (21) used a formula adopted from the self-information of a random variable theorem in order to calculate an IS for labelled molecules (36,37). The formula proved to be empirically appropriate for representing AM profiles. This IS was used to filter out molecules with low IS prior to analysis (see Supplementary Information Supplementary Figures S1–S5).

## RESULTS

### Phage genomes labelled with M.TaqI exhibit a unique and consistent continuous barcode

**Figure 3.**
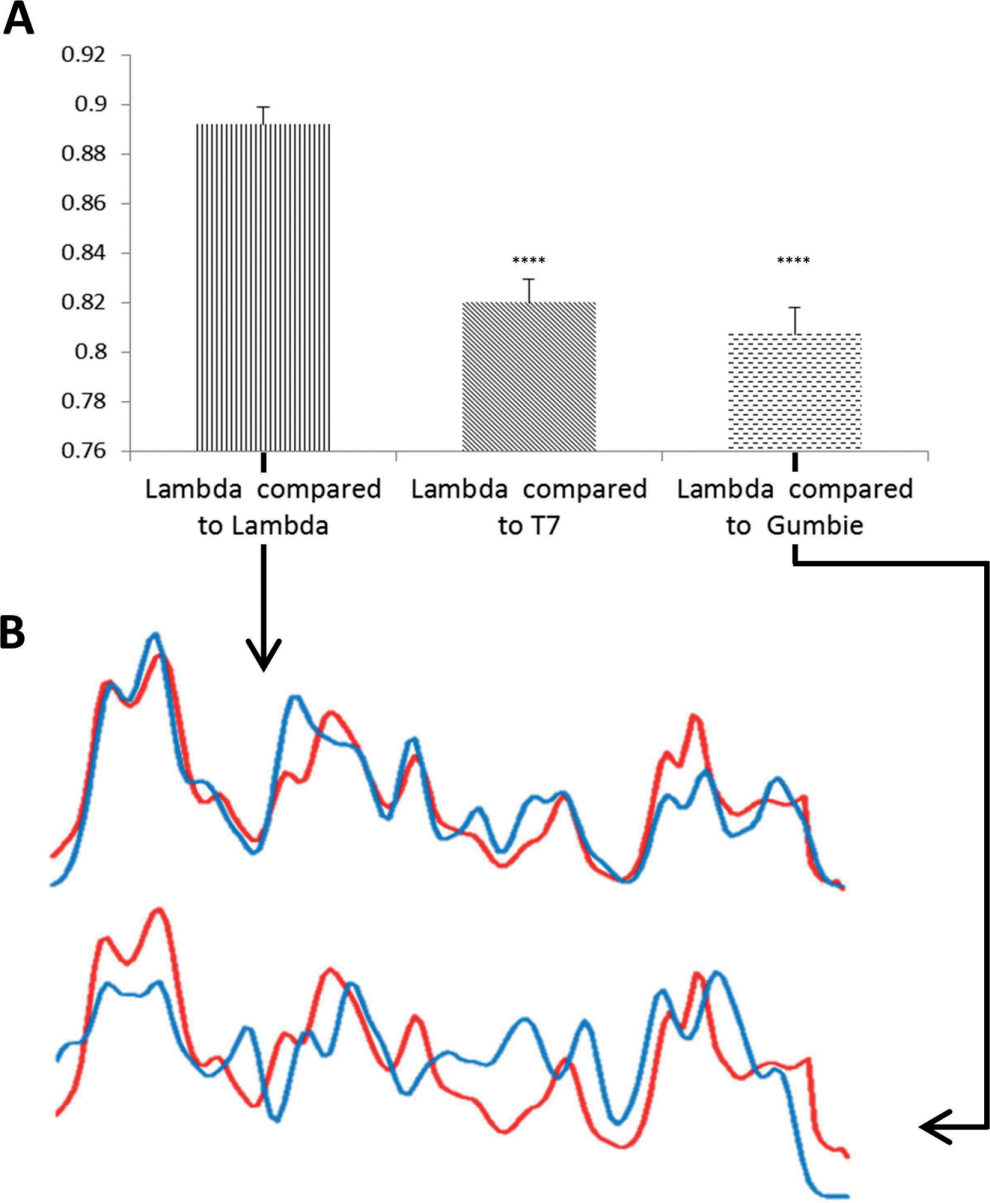
(**A**) AM profiles were generated from λ genomes labelled with AdoYnTAMRA using M.TaqI (27 molecules). Cross correlation was calculated between the λ genomes and theoretical profiles of λ, T7 and GUmbie phage genomes. The CC score was significantly higher when the λ genomes were compared to the theoretical AM profile of λ (*P*-value <0.0001 when compared to T7 and *P*-value <0.0002 when compared to GUmbie paired *t*-test). (**B**) AM profile extracted from an image of one representative λ genome, labelled with M.TaqI and AdoYnTAMRA, when compared to the theoretical AM profile of λ (upper plot, CC value = 0.89) and to the theoretical AM profile of GUmbie (lower plot, CC value = 0.64). In both plots the red profile represents the measured AM from the labelled molecule and the blue profile represents the theoretical AM.

In order to generate sequence specific labels we employed the S-adenosyl-L-methionine (AdoMet)-dependent DNA MTase M.TaqI (TCGA recognition site). The enzyme was provided with a synthetic cofactor AdoYnTAMRA in which the methyl group is replaced by a linker-bound TAMRA fluorophore (see ‘Materials and Methods’). This results in covalent attachment of the TAMRA fluorophore onto adenine within the enzymes recognition sequence (TCGA, Scheme 1). M.TaqI and the synthetic cofactor were used to label the genomes of λ- and T7-phages. The labelling reaction resulted in continuous AM profiles along the stretched genomes that were highly consistent among different molecules in the imaged sample (Figure 2B and Supplementary Figure S10.). To verify that these observed AM profiles are indeed unique and strain dependent, the AM profiles of 27 labelled λ-phage genomes were compared to the theoretical profile calculated from their known sequence. These AM profiles were also compared to the theoretical profiles of the T7 and GUmbie phages, which served as control (due to their length similarity). When compared to its true theoretical profile the CC values are significantly higher than when compared to the false references (*P*-value <0.0001 Figure 3A). Same analyses were done using data acquired from T7 with similar results (Supplementary Figure S7, Supporting Information).

In previous studies, the analysis of AM was done using kymographs which are generated from time lapse recordings of the molecules (consisting of 50–500 frames) (18). Kymographs indeed improve data quality but reduce throughput. In our experiments, it was possible to work with single frame snap shots, instead of kymographs due to the high contrast gained from our labelling method. This significantly shortens acquisition time and increases the throughput of the experiment, a key feature for future large volume screening applications (Figure 2A).

### Strain typing of bacteriophages

**Figure 4.**
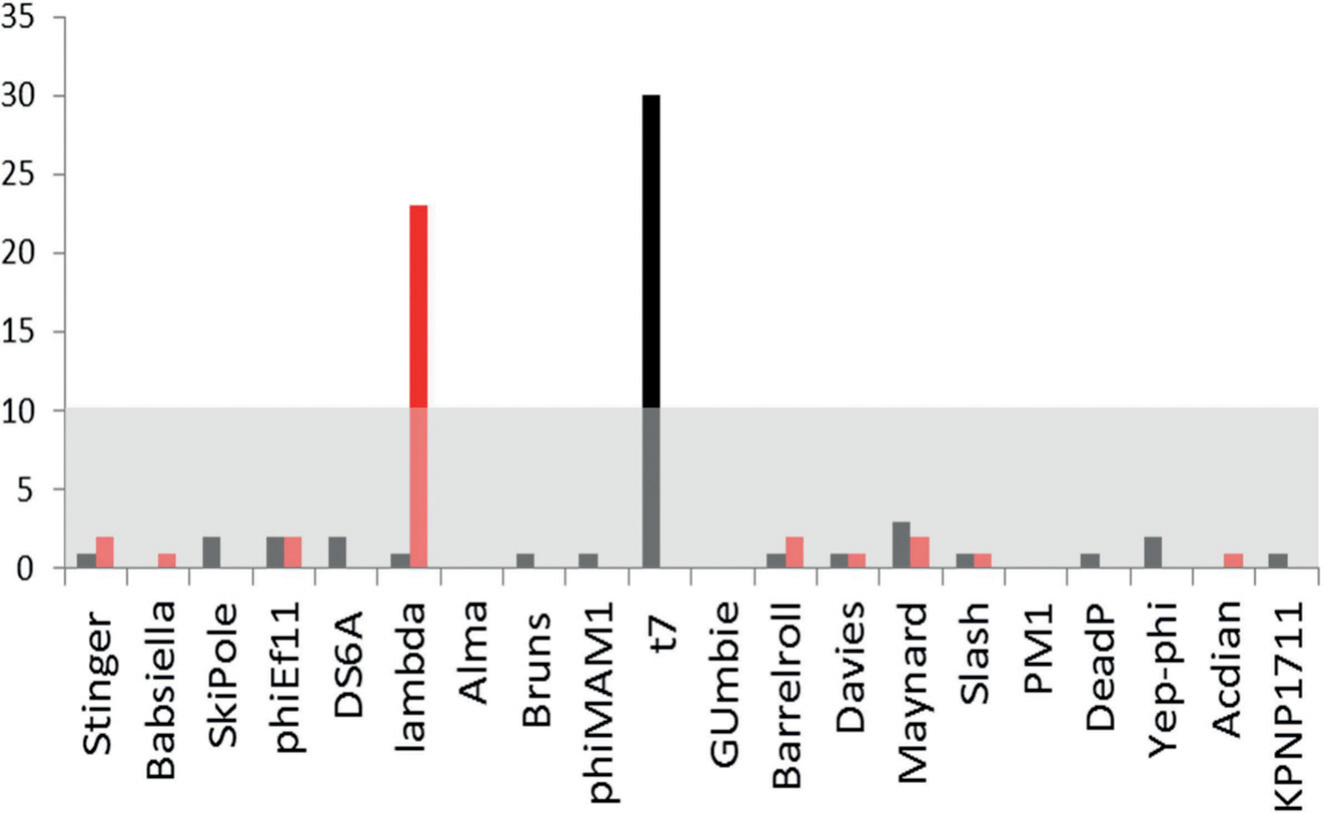
AM from 35 λ genomes, labelled with M.TaqI and AdoYnTAMRA, were compared, using CC, to 20 different phage genomes (all in the length scale of the λ genome, for full list see supporting information). Each labelled genome was affiliated to the phage to which its comparison yielded the highest CC score. The histogram shows the numbers of genomes affiliated to each phage (red bars). Similar analyses were done with 50 labelled T7 genomes (black bars). The grey area in the graph includes all values smaller than the average number of molecules affiliated to a phage, plus one STD of this average.

In order to test whether the experimental AM profiles are sufficiently unique to be used for strain typing of unknown samples, we analysed the data against a reference library with multiple different phages. The genome sequence of 20 random phages (all in the same length scale, for a detailed list see supplementary information Supplementary Table S1), including the genomes of T7 and λ, were used to generate a theoretical AM profile to which the experimental data was compared. We calculated the CC-value for each labelled molecule against all 20 genome profiles and the molecule was classified according to the genome to which the highest CC-value was obtained. The results are depicted in the histogram presented in Figure 4. Distinct peaks containing 66 and 60% of the λ and T7 molecules respectively are clearly visible above the false classification noise. This distribution allows filtering of the data by setting a threshold for accepted results. We calculated the mean and standard deviation of the histogram to find that the number of DNA molecules that were correctly classified was much higher than the value of the mean plus one STD, indicating that reliable classification could be achieved (Supplementary Figures S8 and S9).

### Measuring the information contents of AM profiles

Analysis of an AM profile is based on detection of the modulations in intensity amplitude along the molecule. Therefore the amount of information in the AM profile does not depend linearly on the number of fluorophores, as is the case for DNA barcodes composed of isolated spots, but on the number of peaks and valleys and their contrast. We wanted to compare the relative information content between our labelling method and the previously reported methods that generate intercalation based AM (17–18,21).

In methods based on measuring the local AT/GC ratio, labelling is commonly more frequent than the typical label density for M.TaqI. As a result, small intensity modulations would have a minor impact on the modulation depth. In the case of M.TaqI labelled profiles, changes in label density should cause a more significant impact on AM, resulting in sharper profiles and more information per kbp.

To test this hypothesis, we developed an intuitive, simplified model, based on the model presented at Nillson *et al.*, for obtaining a numerical score representing the information content (IC) in a given combination of DNA sequence and labelling method (21). The IC is composed of two components; the number of distinct features (i.e. spatial information component) and the accumulated peak to valley depths along the molecules (i.e. the contrast component). Both these components are calculated relative to the typical experimental noise in the measurements. In addition, we consider the fact that the contribution of the contrast saturates at some point when additional contrast does not contribute to distinguishing between neighbouring features. The detailed procedure for calculating the IC of a given theoretical AM profile is given as supplementary information.

**Figure 5.**
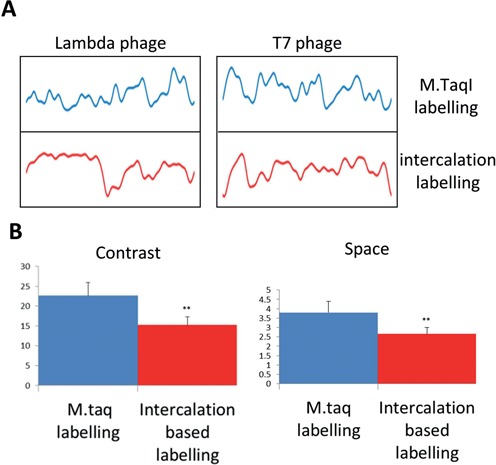
(**A**) Comparisons between the theoretical AM profiles generated by M.TaqI labelling (upper profiles) and intercalation based GC labelling (lower profiles) on λ (left panel) and T7 (right panel) genomes. (**B**) Space and Contrast scores were calculated for 20 different phages (see supporting information for full list). The average contrast score is presented in the left graph and the average space score is presented in the right one. In both, M.taqI labelling (blue bar) shows significantly more information than intercalation based labelling (red bar) (*P*-value <0.005 paired *t*-test).

We calculated the information content for the genome sequence of 20 different strains of phages (for detailed list see the Supplementary Information Supplementary Table S1) using both M.TaqI labelling and intercalation labelling and found that AM profiles generated by M.TaqI labeling, contained on average about 50% more spatial information and about 60% more contrast information compared to intercalation AM profiles which highlights the GC distribution in the sequence (Figure 5B).

## DISCUSSION

Optical mapping of DNA may allow direct and rapid analysis of DNA molecules regardless of preliminary knowledge concerning the sample composition. This feature, combined with the ability to visualize intact phage genomes, makes DNA optical mapping highly attractive for strain typing applications. However, conventional optical mapping approaches, where a genetic barcode is generated via an isolated-label pattern along the DNA molecule, require tailoring of the specific labelling enzyme to the analysed genome sequence in order to guarantee an informative pattern. This is especially true for short genomes, such as those of bacteriophages, where the probability of finding a labelling enzyme that generates a useful pattern of isolated labels drops significantly. For example, the DNA MTase M.BseCI labels DNA within the six base pair recognition sequence ATCGAT. For T7 it generates a pattern of three isolated fluorescence spots along the genome (15). The same enzyme has 15 labelling sites on the λ phage genome, resulting in a semi-continuous fluorescent pattern. Similarly, the commonly used nicking endonuclease Nt.BspQI (GCTCTTC) labels 10 sites on the λ genome and four sites on T7(38). Consequently, the difficulty in finding a labelling enzyme that would be ideal for all different DNA species in mixed samples, limits the strain typing capabilities of short genomes (<150 kbp) due to lack of sufficient unique information in the generated barcodes.

In contrast to classifying DNA molecules based on isolated label patterns, the AM optical mapping used here is based on analysing the pattern of fluorescence modulations generated from dense labelling along the molecule's backbone. The dense labelling implies that substantial modulation of the fluorescence intensity is generated almost regardless of the studied sample. Thus, the AM concept does not require *a priori* knowledge of the sample sequence composition and provides a general labelling scheme for all DNA sequences. In the case of bacteriophages, this labelling strategy offers the opportunity for direct visualization of intact genomes, thus providing coarse grained genetic information on large amounts of DNA in a simple and fast manner (13,39). The straightforward approach allows initial classification of different species from a population into subgroups even without a reference sequence, since genomes are analysed intact, hence avoiding the need for *de novo* assembly. The AM profiles generated are highly informative and enable rapid automated analysis of studied DNA samples based on the degree of similarity between profiles. In this study we introduce a novel method to generate AM profiles by a single step enzymatic reaction where fluorophores are covalently incorporated into the recognition site of M.TaqI. The four base pair recognition sequence of M.TaqI generates patterns that are dense enough for AM analysis. The high stability and contrast of the pattern allowed rapid ‘one-shot’ imaging with no need for averaging. The covalent bond of the fluorophores to the DNA forms a robust pattern and does not require special attention to environmental conditions as is common for earlier techniques based on non-covalent interactions. Furthermore, as we show here, the M.TaqI barcodes contain on average 50–60% more information (in terms of detectable modulations) compared to previous methods, when labelling phage genomes. The distinct differences between the profiles generated by the two labelling schemes (Figure 5A) suggest that it should be advantageous to combine them in a single experiment.

Our labelling scheme was applied to the DNA of two model bacteriophages, λ and T7, and generated continuous unique AM profiles that significantly distinguish the phages from each other. We subsequently used these labelled genomes to simulate a sample with unknown content and were able to detect these phages in a reference library containing 20 phages with DNA of similar length. Despite variations in AM profiles caused by experimental conditions such as labelling efficiency, degree of DNA stretching and dye photophysics, the assay is sufficiently robust to overcome this variability and classify the studied genomes based on their similarity. The method can be directly extended to the analysis of more complex genomes such as bacterial genomes, as is presented for *Escherichia coli* in Supplementary Figure S12.

We propose that labelling of short sequence motifs with M.TaqI, followed by analysis of the modulations in fluorescent amplitude along the DNA, may serve as a general method for DNA identification and analysis. The generality, rapidness and high-throughput this concept offers makes it ideal for strain typing assays.

## SUPPLEMENTARY DATA

Supplementary Data are available at NAR Online.

SUPPLEMENTARY DATA
