# Supplementary material for: Bacteriophage strain typing by rapid single molecule analysis
Source: Nucleic Acids Res. 2015 Oct 10;43(18):e117. doi: 10.1093/nar/gkv563 (PMC4605287; doi:10.1093/nar/gkv563)
Supplement: SUPPLEMENTARY DATA [file supp_43_18_e117__index.html]

Bacteriophage strain typing by rapid single molecule analysis — Bacteriophage strain typing by rapid single molecule analysis — SUPPLEMENTARY DATA 

# Bacteriophage strain typing by rapid single molecule analysis

## SUPPLEMENTARY DATA

- SUPPLEMENTARY DATA
