# Supplementary material for: Bacteriophage strain typing by rapid single molecule analysis
Source: Nucleic Acids Res. 2015 Oct 10;43(18):e117. doi: 10.1093/nar/gkv563 (PMC4605287; doi:10.1093/nar/gkv563)
Supplement: SUPPLEMENTARY DATA [file supp_gkv563_nar-01109-met-g-2015-File008.pdf]

Bacteriophage strain typing by rapid single molecule analysis

Assaf Grunwald[1], Moran Dahan[1], Anna Giesbertz[2], Adam Nilsson[3], Lena K. Nyberg[4], Elmar Weinhold[2], Tobias Ambjörnsson[3], Fredrik Westerlund[4], Yuval Ebenstein[1]*

[1] Raymond and Beverly Sackler Faculty of Exact Sciences, School of Chemistry, Tel Aviv University, Tel aviv, 6997801, Israel
[2]Institute of Organic Chemistry RWTH Aachen University Aachen D-52056 Germany,
[3]Department of Astronomy and Theoretical Physics, Lund University, Sweden,
[4]Department of Biology and Biological Engineering, Chalmers University of Technology, Gothenburg, Sweden

* To whom correspondence should be addressed. Tel: +972-3-6408698; Fax: +972-3- 6405794; Email: uv@post.tau.ac.il

Supplementary information

Evaluating the impact of AM profile information score on data analysis and molecular identification

Considering the computational burden in applications where large experimental data sets are compared to large sets of reference profiles, we seek means for unbiased filtering of the experimental data before analysis. The most computation intensive task is calculating the cross correlation values for each detected molecule against the entire reference set. Thus, filtering the data based on the intrinsic properties of each experimental AM profile before cross correlation may dramatically increase the efficiency and speed of analysis.

Assuming that the more "informative" an AM profile is, the more unique and easy to identify it is, we wanted to estimate the influence of the number of peaks and valleys and their modulations on the analysis of AM profiles. We consider two parameters: *space*, which represents the overall number of peaks and valleys in an AM profile (each peak-valley pair is counted as one) and *contrast*, which is the sum of peak to valley depths along the molecule profile, in units of the noise standard deviation (STD, as determined from the noise levels in our experimental data). To estimate the space contribution we generated a data set containing profiles with varying number of peaks. The data was based on an experimental AM profile containing a single peak which was multiplied *in-silico* to create a data set containing profiles of increasing numbers of peaks and valleys (Figure S1. A.). As expected, there is a linear dependency between the number of peaks and the information score, which was calculated using the analysis program (Figure S1. B.) (1–4). The cross correlation value on the other hand is insensitive to the number of features in the profiles. Cross correlation values scale only with the degree of similarity between the reference and the data. Consequently, although a molecule displaying many peaks and valleys is most likely more unique, it will yield the same cross-correlation value as a molecule displaying only two peaks if both molecules are compared to their corresponding reference profiles.

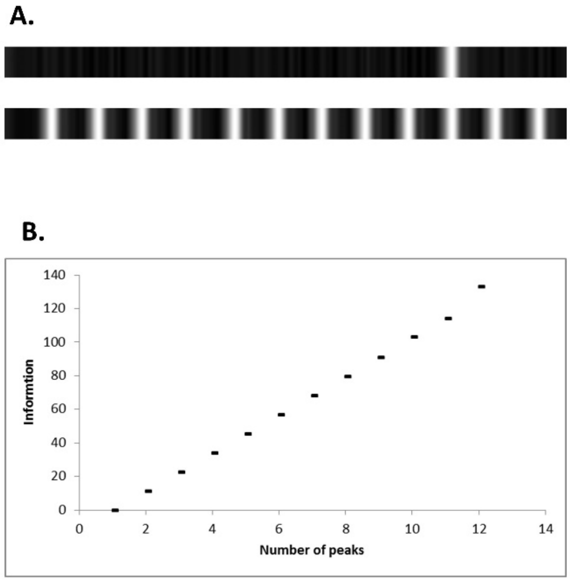

Figure S1. **A.** Representative images from the data set we used to examine the impact of the number of peaks and valleys in AM profiles on the information score. The upper image contains 1 peak and the lower contains 12. **B.** A plot demonstrating the dependency between the information score (Y axis) and the number of peaks (X axis).

To evaluate the contrast contribution we generated a data set of AM profiles, each containing two peaks with increasing intensities. The first profile in the set was the experimental noise, representing a signal to noise (SNR) ratio of 1. In the following profiles SNR was increased gradually by adding one noise STD unit to the peak intensity for each subsequent profile. Next, we used the analysis software to calculate the information score for each contrast level and study the relation between image contrast and information values (Figure S2. A.). In addition, cross-correlation was calculated with a theoretical profile corresponding to the generated data and the cross-correlation value was plotted against the number of noise STD units added to the peak intensities (Figure. S2. B.). We find that the information score increases gradually with increasing contrast. However, for the cross correlation tests we see a strong increase in correlation that reaches saturation at contrast values of about 7 STDs above noise level.

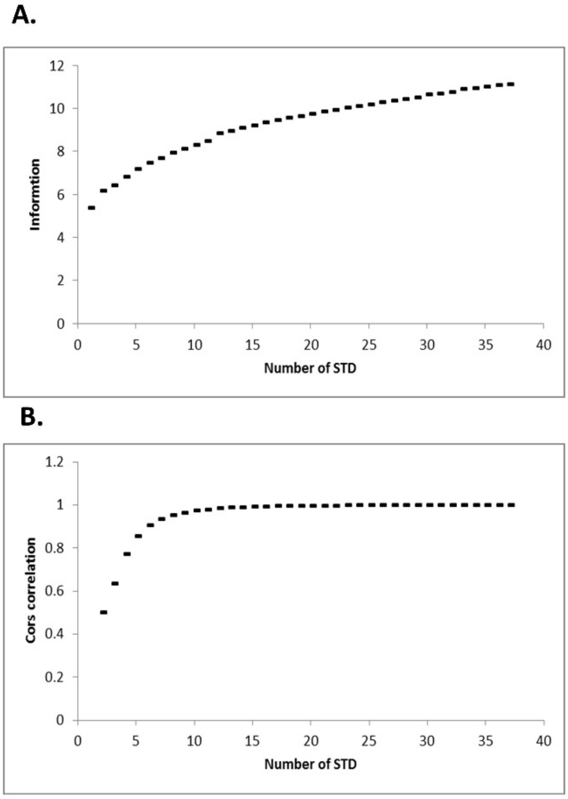

Figure S2. **A**. A plot demonstrating the dependency of the information score on the image contrast (displayed in units of noise STD above the noise level) **B**. A plot demonstrating the dependency between the cross correlation score (Y axis) and the number of STD levels of the peak in the profile above the noise level (X axis).

From these analyses we conclude that the number of modulations in the intensity profile of a molecule (the space component) has a strong impact on the information content (representing the uniqueness of the profile). The contrast value, on the other hand, has a rather weak impact on the information score but a strong impact on the cross correlation score at low SNR values. This impact saturates above a level where additional contrast does not contribute any further to the fit (signal levels of above of 7 noise STDs in our case).

We also wanted to check the influence of DNA length on the information score in our experimental data, as reducing length results in elimination of peaks and valleys and thus reduce information. Typical AM profiles, generated from labelled λ genomes were gradually truncated (reducing a 5 kbp long tail at a time). Information analysis showed that for these molecules the threshold length for the information criteria of IS=80 was ~35-40 kbp (Figure, S3). It is important to emphasize that these results are typical for λ genomes and other sequences might have different label distributions and thus different length thresholds.

A.

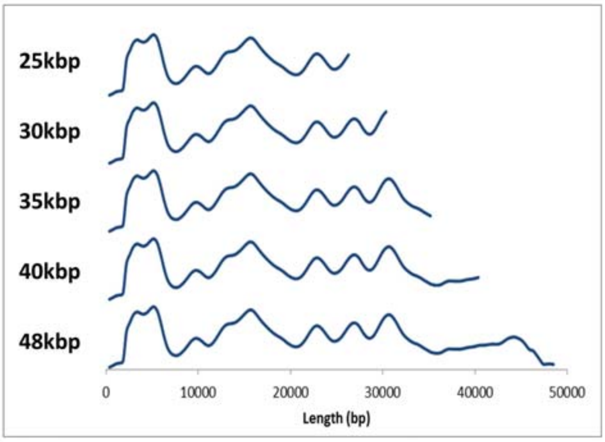

B.

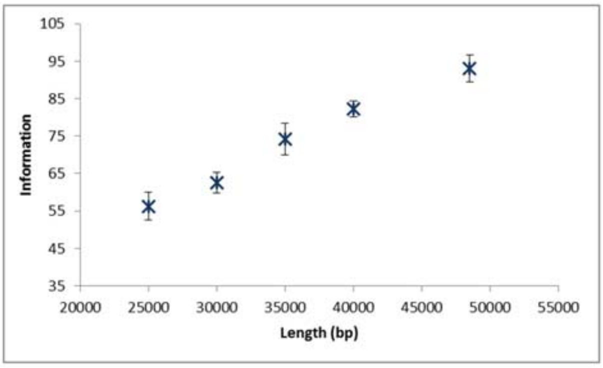

Figure S3. **A.** Typical AM profile of labelled λ molecule was truncated into fragments of 48.5 (full length), 40, 35, 30, and 25 kbp. Fragment plots are presented with corresponding lengths to their left. **B.** Information scores from 5 truncated λ genomes were calculated for each fragment (48-25 kbp) and the average score was scatter plotted against its corresponding length (blue markers, error bars represents standard errors values).

Effect of false labelling on cross correlation values

False or missing peaks along the AM profile may be the result of absence/excess of fluorescent labels, or due to problems in the imaging procedure. To estimate the effects of false peaks on the goodness of fit to the theoretical data we generated an artificial data set containing profiles with 2-12 peaks. In this data set the two extreme peaks were kept constant and peaks from the middle of the profile were reduced one at a time resulting in a profile with a constant length and varying number of modulations (Figure S4.A). We used a profile containing 6 peaks as the reference and calculated its cross correlation with all other profiles (2-12 peaks) in order to simulate both false positive and false negative labelling events (Figure S4. B.). The cross correlation score decreases rapidly with both additional

peaks (false positive) and missing peaks (false negative), with false negatives having a slightly larger effect on the correlation. These results emphasize the ability of the method to distinguish the correct patterns from mislabelled patterns based on CC analysis.

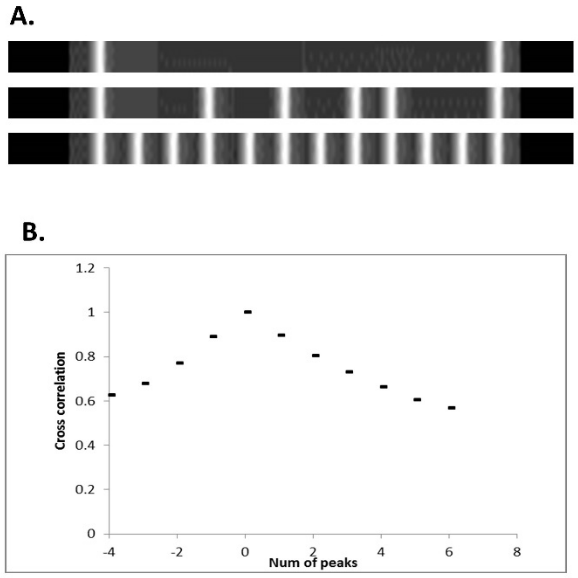

Figure S4. **A.** Representative images from the data set we used to examine the impact of false labelling on CC. The upper image contains 2 peaks, the middle contains 6 (this profile served as reference to which all profiles were compared) and the bottom contains 12 peaks. **B.** A plot demonstrating the relation between the CC score (Y axis) and the number of peaks (X axis). The X axis values are the number of additional / missing peaks relative to the 6 peak reference.

Calculation of the information content value for a given theoretical AM profile

Based on our observations above, we developed an intuitive method to estimate the information contents (IC) for a given DNA sequence and labelling method. First we generate a theoretical AM profile from the known sequence, considering the distribution of labelling sites (depending on the labelling method), the point spread function of our microscope and the experimental stretching factor of the molecules(1–3). Next, we calculate the noise STD (normalized to percentage of the mean signal) from our experimental data.

Using these, we count the number of distinct features along the AM profile (a feature is defined as distinct if the intensity difference between a peak and a valley is larger than 3 STDs). The number of peak and valley pairs along the profile composes the spatial modulation component of the information score. The modulation depth/contrast component is the sum of STD units in all peak to valley differences, where we have set the saturation value at 7, based on our observations above. We used this IC score calculation in order to assess the effect of the information content on data analysis and to compare

between our new labelling method and intercalation based approaches (see results). In principle, this intuitive approach to calculate information content yields results that are similar in nature to the previously reported information score (IS) calculated based on the self-information of a random variable (5, 6). The latter is calculated automatically by our analysis software and both calculation methods were used during data analysis.

Data filtering

During data analysis we wished to filter out "bad" molecules from our experimental data. We first filtered out molecules that where shorter than 75% of the expected length. This filtering step was aimed to filter out fragmented molecules and can be skipped when studying samples of unknown content.

In addition, we only used molecules with IS higher than 80 and a cross correlation score higher than 0.85 (Figure S5.), when compared to any of the reference sequences (simply put, if the molecule is compared to the multi sequence reference library, the best score must be higher than 0.85).

It is important to emphasize, that the IS for experimental data is determined by the analysis software and may vary according to the imaging parameters, stretching factor and quality of labelling.

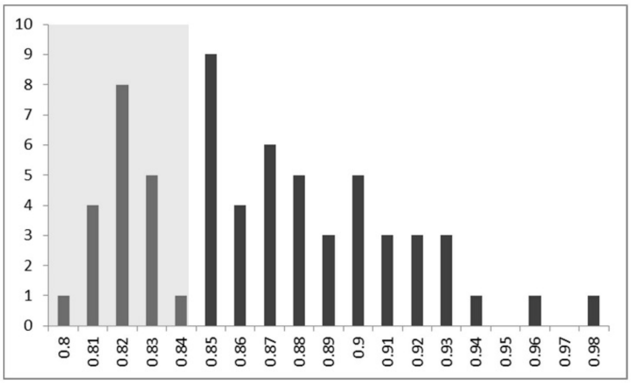

Figure S5. A histogram representing the distribution of CC scores of AM generated from labelled λ genomes fitted to their best fit genome in the theoretical reference library. The grey area of the histogram highlights the molecules that had CC scores lower than the threshold value. 35 out of 63 molecules (~56%) had a CC score higher than our threshold value of 0.85.

Imaging parameters

To allow sensitive detection of the intensity modulations along the molecules it was crucial to take advantage of the full dynamic range of the camera. For this experiment we used the Hamamatsu Orca Flash 4 sCMOS rather than the Andor EMCCD due to its higher dynamic range. This is important due to the fact that intensity along a molecule may range between single molecule fluorescence generated by an isolated label and intense fluorescence from a dense cluster of molecules. Imaging conditions such as excitation intensity and camera integration time were optimized to prevent saturation of the fluorescence signal but still

collect signal from the sparsely labelled dark regions along the DNA. The 532nm laser radiation density was ~9.3 mW/cm^{2}and images were recorded at a frame rate of 250 ms with 2X2 binning (equivalent to a 1024 x 1024 array with 13 µm pixels). Analysis was performed on molecules under uniform excitation (<15% variation) in order to account for non-uniformities in the excitation field.

Estimating the efficiency of the M.TaqI AdoYnTAMRA labelling reaction

To estimate the efficiency of the labelling reaction and to find out the amount of enzyme needed for full labelling of all its recognition sites, we used a protection assay. In this assay a fixed amount of DNA is modified with varying amounts of enzyme (reduced by half between different reactions). After modification all samples are incubated with the restriction enzyme R.TaqI, which has the same recognition sequence as the modifying enzyme but does not digest modified sites. Afterwards, all the samples are loaded and run on an agarose gel. In the case of full labelling only one band, of non-restricted DNA is expected, but if the modification is partial, several lower bands should be seen. We performed this assay with λ DNA, M.TaqI and AdoYnTAMRA cofactor starting with an amount of 1 equivalent of enzyme (one enzyme molecule per each M.TaqI site) and found that even at 1/64 equivalents of enzyme to labelling sites, DNA is fully protected against restriction. We conclude that the labelling reaction approaches 100% efficiency at higher M.TaqI concentrations (Figure S6). In order to ensure full modification of all sites our experiments were performed at an excess of ~6 equivalents of M.TaqI (100-fold excess).

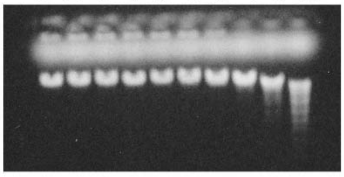

Figure S6.A protection assay was performed on λ DNA using M.TaqI and AdoYnTAMRA. After modification, samples were challenged with the restriction enzyme R.TaqI and run on an agarose gel. The DNA is stained with GelRed while the TAMRA fluorophor of the cofactor is also visible as diffuse fluorescence in all lanes above the DNA. In lane 1.one equivalent of enzyme was used and its amount was reduced by half in each of the following 9 lanes. It is clearly seen that the entire DNA in samples 1-7 is fully protected, while samples 8-10 exhibit reduced protection. This assay shows that even when ~2% of 1 equivalent is used the efficiency of the labelling reaction approaches 100%.

T7 genomes labelled with M.TaqI exhibit a unique barcode

To verify that the AM profiles generated from T7 genomes labelled by M.TaqI are indeed unique and strain dependent we performed similar analysis as we did with the λ data (Figurer 3.A.). We compared AM profiles of 29 labelled T7 genomes to the theoretical profile calculated from their known sequence. We also compared it to the theoretical AM profiles of the λ and GUmbie phages, which served as control (due to their length similarity). We found that when compared to its true theoretical AM profile the CC values are significantly higher than when compared to the false references (P-value <0.0001, Figure S7).

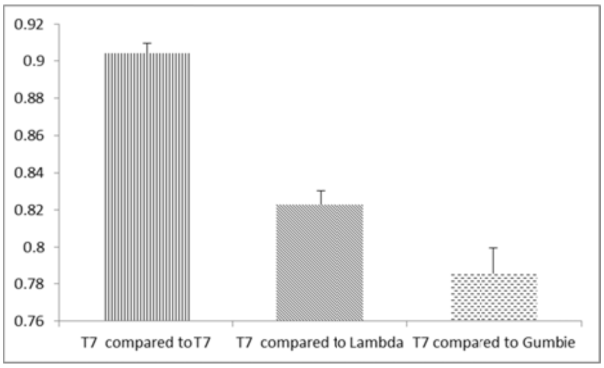

Figure S7. AM profiles were generated from T7 genomes labelled with M.TaqI (29 molecules). Cross correlation was calculated between the AM profiles and theoretical profiles of λ, T7, and GUmbie phage genomes. The CC score was significantly higher when the T7 genomes were compared to the theoretical AM profile of T7 (P-value <0.0001 paired t-test).

Cross correlation analysis between AM profiles from λ DNA labelled with M.TaqI and all 20 theoretical sequences of phages in the reference library.

Cross correlation analysis was performed between AM plots of λ DNA labelled with M.TaqI and all 20 phage sequences in the reference library (as shown in detail in Figure 3.A &B for only 3 sequences). The average CC score for false fits of λ data to the other 19 phage genomes was ~0.8 with a standard error of ~0.01. We defined the CC threshold for a reliable fit to be the average CC plus 5 standard error units. The CC score of the data fitted to λ was higher by more than 8 standard error values than the averaged CC value of all other 19 phages and also showed statistical significance (Figure S8).

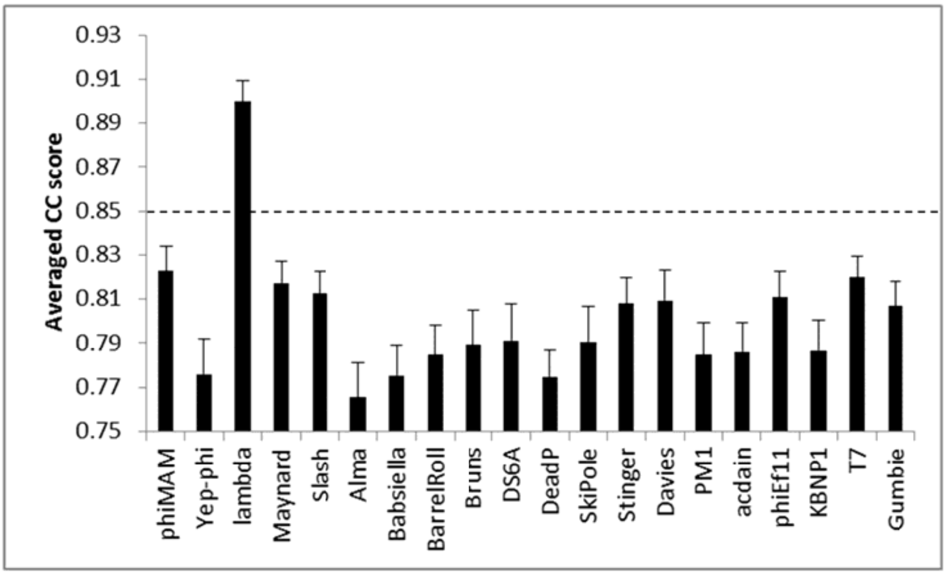

Figure S8. AM profiles were generated from λ genomes labelled with M.TaqI (14 randomly chosen molecules). Cross correlation was calculated between the AM profiles and theoretical profiles of all the phage genomes in our reference library (table S1). The average CC score was higher when the λ genomes were compared to the theoretical λ profile than the averaged CC scores when aligned to the other phages (P-value ranging between 0.00013 and 0.000002 paired t-test). Dashed line represent to threshold value of 0.85 for a good comparison.

Correlation between nucleotide identity and CC value

In order to check the degree of nucleotide identity between all the reference sequences in our library and the λ sequence we used BLASTn from NCBI, which is optimized for sequences that are similar but not identical and can tolerate composition differences between compared sequences (7). The average identity score for the false pairs was 385 while λ compared to itself results in a score of ~88,000. The BLASTn scores were plotted against the corresponding CC scores in order to assess the correlation between these measures (Figure S9).

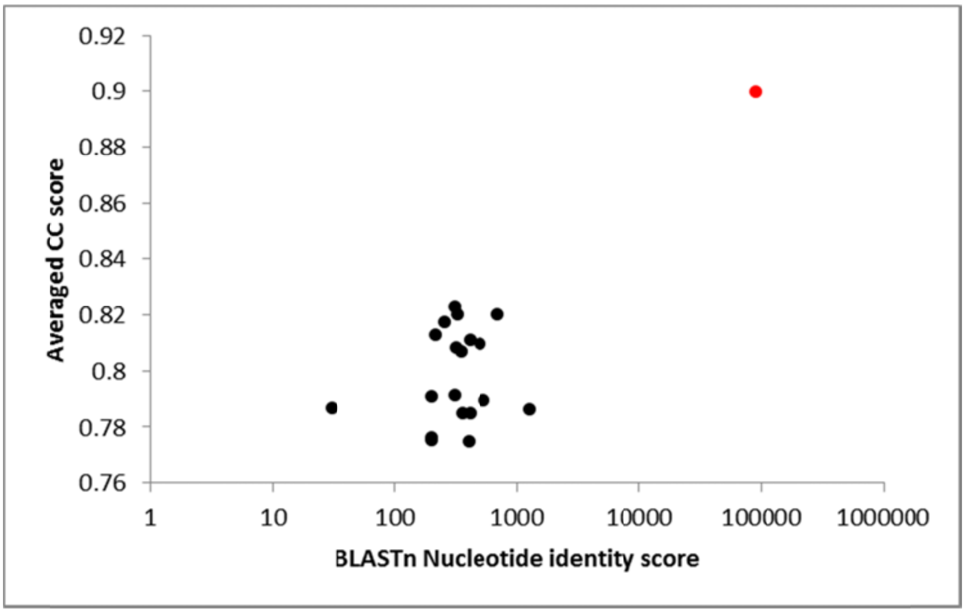

Figure S9. Nucleotide identity scores were calculated between all the phage sequences in our library and the λ sequence using BLASTn. These scores were co-plotted against the average CC scores of λ AM profiles and the various reference sequences (calculated in Figure S7). The nucleotide identity scores are presented on a logarithmic scale. The black markers represent λ AMs compared to all other 19 sequences. The red marker represents λ when compared to itself, which exhibits distinct differences from the comparison of all other sequences in both measures.

Plots of AM profiles generated from λ molecules labelled with M.TaqI

We plot 5 representative AM profiles obtained from the λ data. CC analysis is able to correctly classify these molecules despite the noticeable variation in the AM profile details.

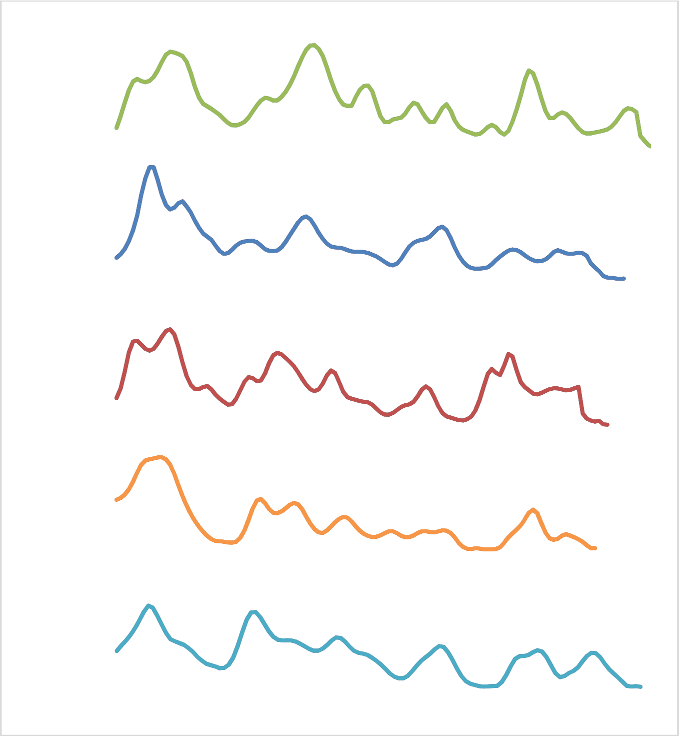

Figure S10. AM profiles of λ molecules labelled with M.TaqI are co-plotted to exhibit their similarity.

Cross correlation analysis of AM profiles from λ DNA labelled using an Intercalation based method

The analysis presented in Figure 3 in the main text was performed also on intercalation based data in order to directly compare between the two labelling methods. Using the intercalation data, we calculated CC values between experimental λ AM plots and the theoretical plots of λ, T7 and Gumbie phages. This analysis shows that the average CC difference between true and false fits is smaller to that presented in Figure 3. Furthermore, due to the high noise in the intercalation based measurements, the error bars for the calculated CC averages are much larger and result in difficulty to significantly distinguish between true and false fits (Figure S11). It is important to note that due to the noisy data generated by intercalation, previous published work used time averaging in order to obtain smoother AM profiles. Here, for the sake of comparison, we used AM profiles generated from a single image, identical to the analysis of the M.TaqI data.

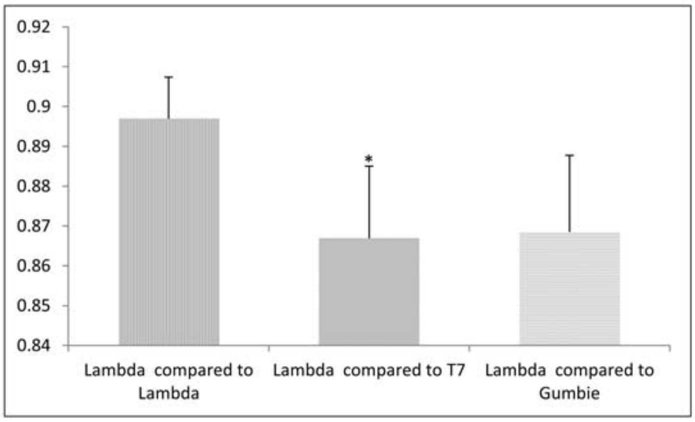

Figure S11. AM profiles were generated from λ genomes labelled by an intercalation based method (1) which highlights their GC rich regions (13 molecules) (1, 3, 4). Cross correlation was calculated between the AM profiles and theoretical profiles of λ, T7, and GUmbie phage genomes. The CC score was higher when the λ genomes were compared to the theoretical AM profile of λ. However the values show high variability when compared to T7 and Gumbie. A statistical paired t-test between the true and false fits yielded low significance for T7 (P-value >0.015) and no significance when compared to GUmbie (p-value>0.06).

Preliminary data for *E. coli* AM profiles

In order to demonstrate the generality of the M.TaqI AM approach we have performed a similar experiment with E. coli genomic DNA. The DNA was extracted from bacteria, labelled and imaged exactly as the T7 and λ DNA. The experiment resulted in genomic fragments with an average length of ~100 kbp and distinct AM profiles (Figure S12). These fragments are about 2 times longer than the phage genomes we tested and thus should exhibit higher information contents and display accurate assignments to the reference sequence. This will be tested thoroughly in a follow-up project.

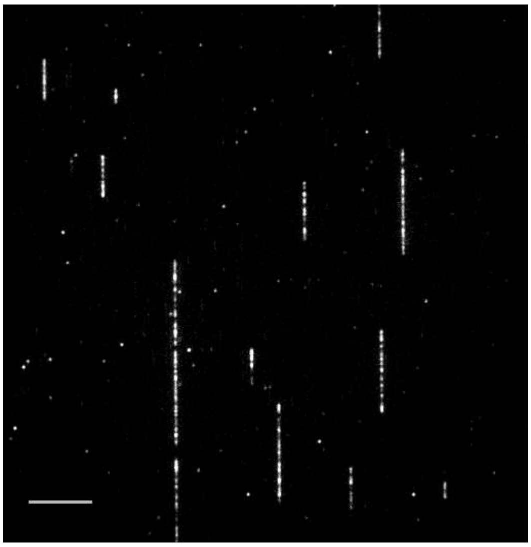

Figure S12. An image of a field of view of the nanochannels containing stretched and labelled fragments of E.coli genomes. The scale bar at the bottom left corner represents a distance of 10 µm.

List of phages used as a reference library

We used a reference library containing 20 different phages both for our information analysis and for the strain typing analysis, the phages names and their length in kbp are given in table S1:

Phage's name	Phage's length (kbp)
Mycobacterium phage Stinger	69.6
Mycobacterium phage Babsiella	48.4
Mycobacterium phage SkiPole	53.1
Enterococcus phage phiEf11	42.8
Mycobacterium phage DS6A	60.6
Enterobacteria phage λ	48.5
Mycobacterium phage Alma	53.2
Mycobacterium phage Bruns	53
Serratia phage phiMAM1	157.8
Enterobacteria phage T7	39.9
Mycobacterium phage GUmbie	57.4
Mycobacterium phage BarrelRoll	59.7
Paenibacillus phage Davies	48.5
Salmonella phage Maynard	45.6
Bacillus phage Slash	35.2
Pectobacterium phage PM1	55.1
Mycobacterium phage DeadP	56.5
Yersinia phage Yep-phi	38.6
Mycobacterium phage Acadian	69.9
Escherichia phage KBNP1711	76.2

REFERENCES

1. Nyberg,L.K., Persson,F., Berg,J., Bergström,J., Fransson,E., Olsson,L., Persson,M., Stålnacke,A., Wigenius,J., Tegenfeldt,J.O., *et al.* (2012) A single-step competitive binding assay for mapping of single DNA molecules. *Biochem. Biophys. Res. Commun.*, **417**, 404–8.

2. Noble,C., Nilsson,A.N., Freitag,C., Beech,J.P., Tegenfeldt,J.O. and Ambjörnsson,T. (2013) A Fast and Scalable Algorithm for Alignment of Optical DNA Mappings. *Quant. Biol.*

3. Nilsson,A.N., Emilsson,G., Nyberg,L.K., Noble,C., Svensson Stadler,L., Fritzsche,J., Moore,E.R.B., Tegenfeldt,J.O., Ambjörnsson,T. and Westerlund,F. (2014) Competitive binding-based optical DNA mapping for fast identification of bacteria - multi-ligand transfer matrix theory and experimental applications on Escherichia coli. *Nucleic Acids Res.*, **42**, E118.

4. Reisner,W., Larsen,N.B., Flyvbjerg,H., Tegenfeldt,J.O. and Kristensen,A. (2009) Directed self-organization of single DNA molecules in a nanoslit via embedded nanopit arrays. *Proc. Natl. Acad. Sci. U. S. A.*, **106**, 79–84.

5. Cover,T.M. and Thomas,J.A. (1991) Elements of Information Theeory. Chapter 2 Entropy , Relative Entropy and Mutual Information.

6. Azbel',M.Y. (1973) Random Two-Component One-Dimensional Ising Model for Heteropolymer Melting. *Phys. Rev. Lett*, **31**, 589–592.

7. Johnson,M., Zaretskaya,I., Raytselis,Y., Merezhuk,Y., McGinnis,S. and Madden,T.L. (2008) NCBI BLAST: a better web interface. *Nucleic Acids Res.*, **36**, W5–9.

.
